# Insoles Treated with Bacteria-Killing Nanotechnology Bio-Kil Reduce Bacterial Burden in Diabetic Patients and Healthy Controls

**DOI:** 10.1155/2018/7678310

**Published:** 2018-06-28

**Authors:** Difei Lu, Xiaohui Guo, Yun Li, Bo Zheng, Junqing Zhang

**Affiliations:** ^1^Department of Endocrinology, Peking University First Hospital, Beijing, China; ^2^Institute of Clinical Pharmacology, Peking University First Hospital, Beijing, China

## Abstract

Our study investigated the effectiveness of bacteria-killing nanotechnology Bio-Kil socks on bacterial burden reduction in diabetic patients and healthy individuals. Four strains of *S. aureus* and four strains of *E. coli* were cultured and dropped on Bio-Kil socks and control socks for 0 h, 8 h, and 48 h of incubation. Diluted samples were inoculated and bacterial counts were recorded. Additionally, 31 patients with type 2 diabetes and 31 healthy controls were assigned to wear one Bio-Kil sock on one foot and a control sock on the other for four hours, and then they were told to exchange socks from one foot to the other for four hours. The socks were sampled and diluted and then inoculated to record bacterial counts. Bacterial counts were reduced in Bio-Kil socks compared with control socks in all *S. aureus* strains after 0 h, 8 h, and 48 h of incubation. In *E. coli* strains, bacterial counts declined in Bio-Kil socks comparing with control socks in most of the experiments with ESBL-negative *E. coli* and ATCC35218 at 0 h and 48 h of incubation. In all participants, the mean bacterial counts significantly decreased in Bio-Kil socks in comparison with control socks both at 0 h and at 40 h of incubation (*p* = 0.003 at 0 h and *p* = 0.006 at 40 h). Bio-Kil socks from diabetic patients showed significantly lessened bacterial count at 40 h of incubation (*p* = 0.003). In healthy individuals, Bio-Kil socks reflected a significantly smaller mean bacterial count than control socks (*p* = 0.016). Socks using Bio-Kil nanotechnology efficiently reduce bacterial counts in both diabetic patients and healthy individuals and might exert stronger efficacy in Gram-positive bacteria.

## 1. Introduction

A diabetic foot is a severe complication of diabetes, which brings about high disability and mortality rates, as well as social-economic burden on patients with diabetes. The associated foot problems include ulceration, infection, and amputation and have a tremendous impact on health-related quality of life for diabetic patients. Yearly incidence of foot ulcers in diabetic patients is estimated as 2%, and its recurrence rate is as high as 30% to 40% in the first year after successfully healing from a foot ulcer [1]. Among all diabetes-associated foot problems, 80% of them are preventable [2]. Since the prevention of diabetic foot plays a critical role in management of foot problems, new methods for foot ulcer prevention such as insoles are emerging and proved their efficacy in clinical trials. In a randomized controlled trial (RCT), custom-made foot orthoses showed a significant reduction in plantar pressure [3]. Another custom-made footwear focused on peak pressure reduction, but the primary end-point ulcer recurrence was not significantly declined [4]. A nanotechnology sock, which was designed to increase skin moisture content, displayed protective function in subjects with diabetes [5].

Bio-Kil (Cargico Group, Taiwan) is a bacteria-killing nanotechnology agent comprising inorganic metal components and organic quaternary ammonium compounds (QACs) [6]. Bio-Kil molecules could attract pathogens due to a high-affinity structure and electric field and damage the membrane structure of microorganisms with its electrical charge to kill the pathogens. Furthermore, the bacterial killing efficacy of Bio-Kil agent remains even after over fifty times of washing as a result of the permanent, covalent bond between Bio-Kil agents and the surface of the textile fibers [7]. The Bio-Kil antibacterial catalyst was applied to the ICU environment and surface of instruments such as bed sheets, pillows, computer keyboards, nursing station desktops, and surfaces close to patients, where it was proved to maintain a long-term bactericidal function [8]. However, it was the first time that Bio-Kil nanotechnology was being applied to footwear for patients with diabetes and healthy individuals. The possible bactericidal effect on the local foot area was investigated in the following study that we conducted, thereby providing a choice for patients with at-risk diabetic foot or foot ulcer to suppress bacterial burden in their feet.

## 2. Material and Methods

### 2.1. Bacterial Strain Experiments

Four clinical separated bacterial strains and four standard bacterial strains were provided by the Institute of Clinical Pharmacology, Peking University First Hospital: two strains of methicillin-resistant *Staphylococcus aureus* (MRSA) (15B183 and ATCC43300), two strains of methicillin-sensitive *Staphylococcus aureus* (MSSA) (15B190 and ATCC29213), two strains of extended spectrum *β*-lactamase- (ESBL-) positive *Escherichia coli* (E. *coli*) (15B254 and ATCC35218), and two strains of ESBL-negative E. *coli* (15B253 and ATCC25922). S. *aureus* strains were cultured in Tryptone Soya Broth (TSB) (Oxoid, UK) and E. *coli* strains were cultured in Luria Broth (LB) (Oxoid, UK) at 35°C for 16 hours. Bio-Kil nanotechnology socks (Cargico Group, Taiwan) in grey color and custom-made control socks in white color, which were designed to be similar in appearance and textile with Bio-Kil socks, were cut into 1 cm × 1 cm squares. 100 *μ*l of each bacterial strain culture was dropped on Bio-Kil sock and control sock squares. We planned to evaluate immediately, after 8 hours of contacting (imitating 8 hours of working), and a lasting contacting time of 48 hours, and sock squares were incubated separately for 0 h, 8 h, and 48 h. After incubation, sock squares were steeped in 5 ml Mueller-Hinton Broth (MHB) (Oxoid, UK) and vibrated for 2 minutes and then diluted with MHB for 10^2^, 10^3^, and 10^4^ times. 100 *μ*l of each diluted samples was inoculated into Mueller-Hinton agar (Oxoid, UK) with blood for S. *aureus* strains or China blue agar for *E. coli* strains and then placed in a 35°C incubator for 24 hours. The number of colonies was recorded after incubation as colony-forming units (CFUs)/cm^2^.

### 2.2. Experiments in Diabetic Patients and Healthy Participants

#### 2.2.1. Settings

The following study was conducted during the period of 20 September to 20 October 2017 in the Department of Endocrinology, Peking University First Hospital in Beijing, China. The Research Ethics Committee of Peking University First Hospital (PUFH) approved the protocol for the purpose of our study in this manuscript. Written informed consent with signatures was obtained from all participants enrolled in this study. Thirty-one patients who were diagnosed with type 2 diabetes based on the World Health Organization criteria, which included fasting plasma glucose ≥ 7.0 mmol/l, and/or 2-hour plasma glucose ≥ 11.1 mmol/l or random plasma glucose ≥ 11.1 mmol/l. Healthy individuals were defined as no diagnosis of any diseases, for instance, type 2 diabetes, hypertension, and coronary artery diseases. Participants were excluded in the study when subject had existing foot ulcer or history of foot ulcer, foot infection, or was incapable of wearing socks or fully understanding the research protocol. Anthropometric data was collected after enrollment, which consisted of age, sex category, history of diabetes, history of peripheral artery disease, and history of diabetic peripheral neuropathy.

#### 2.2.2. Embedding and Sampling of Socks

Thirty-one diabetic patients and thirty-one healthy individuals were assigned to wear one Bio-Kil sock on one foot and wear a control sock on the other for 4 hours and exchanged bilaterally and wear the socks for another 4 hours. During the process, participants were unaware to which sock Bio-Kil nanotechnology was applied, which was to say a single-blinded study design. After eight hours of wearing, socks were cut into 1 cm × 1 cm squares and were incubated separately for 0 h and 40 h to compare with the incubating time in bacterial strain experiments. Sock samples were steeped in 5 ml MHB after incubation and vibrated for 2 minutes and then diluted with MHB for 10 and 10^2^ times. 100 *μ*l of each samples and diluted samples was inoculated into Trypticase Soy Agar and incubated at 35°C for 24 hours. After incubation, the number of colonies was recorded as colony-forming units (CFUs)/cm^2^.

### 2.3. Statistical Analysis

Data were analyzed using the Statistical Package for the Social Sciences for Windows (SPSS version 16.0, Chicago, IL, USA). Continuous variables were expressed as mean ± standard deviation (SD) and categorical variables were as percentage. Student's paired *t*-test was used for quantitative variables, and non-Gaussian variables were compared using Mann–Whitney *U* test. A *p* value of <0.05 was considered to be of statistical significance.

## 3. Results

### 3.1. Bacterial Strain Experiments

In the experiments with bacterial strains, bacterial counts were reduced in Bio-Kil socks compared with control socks in all *S. aureus* strains and in 0 h, 8 h, and 48 h of incubation, indicating an effective and continuous bacterial killing activity in socks treated with Bio-Kil (Table 1). However, in *E. coli* strains, bacterial counts declined in Bio-Kil socks comparing with control socks in most of the experiments with ESBL-negative *E. coli* and ATCC35218 at 0 h and 48 h of incubation (Table 1). These results indicated that the bactericidal nanotechnology Bio-Kil might exert a stronger bacterial killing effect on Gram-positive bacteria.

### 3.2. Experiments on Diabetic Patients and Healthy Participants

The baseline data of the thirty-one patients with type 2 diabetes and thirty-one healthy individuals was listed in Table 2. There were more female participants in both diabetes group and healthy individual group. The mean age in the two groups was unequal (diabetes versus healthy subjects, 56.6 ± 13.1 versus 28.0 ± 4.5). The duration of diabetes history was 9.5 ± 6.7 years in the diabetes group. Thirteen of the thirty-one diabetic patients (41.9%) were diagnosed with diabetic peripheral neuropathy, and four of them (12.9%) were diagnosed with peripheral artery disease.

**Figure 1:**
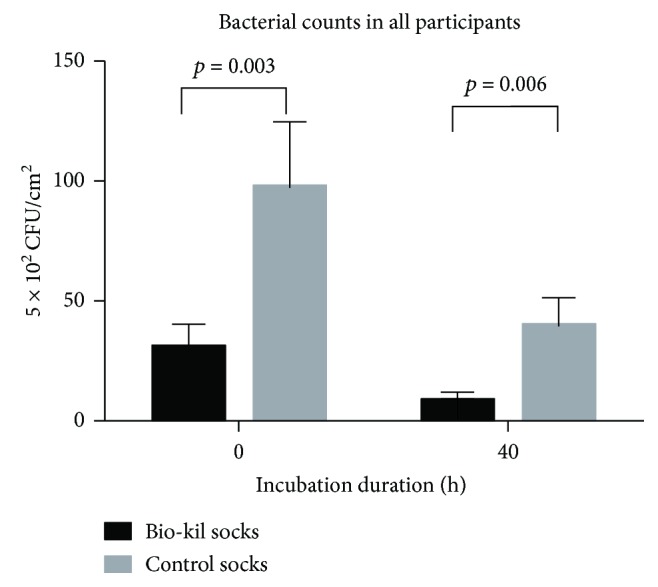
Bacterial counts in Bio-Kil-treated socks and control socks in all participants.

**Figure 2:**
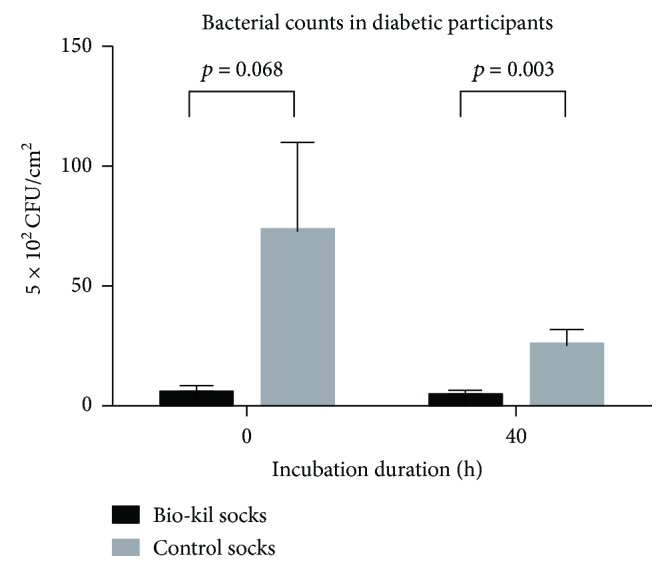
Bacterial counts in Bio-Kil-treated socks and control socks in diabetic patients.

**Figure 3:**
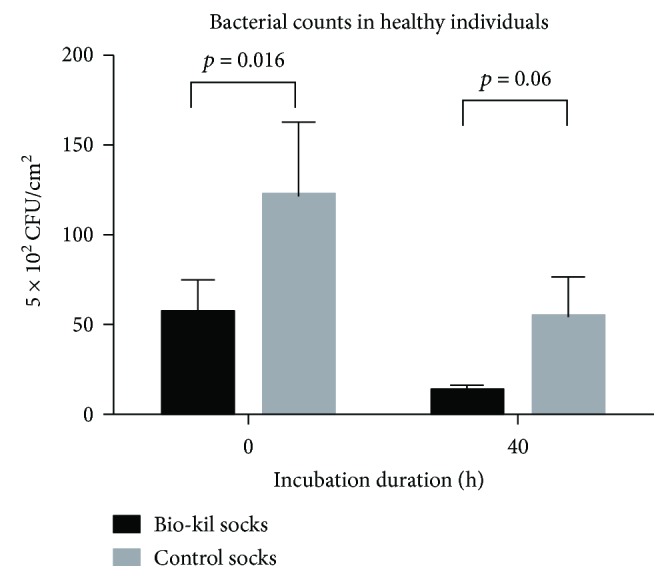
Bacterial counts in Bio-Kil-treated socks and control socks in healthy individuals.

In all participants, the mean bacterial counts significantly decreased in Bio-Kil socks in comparison with control socks both at 0 h and at 40 h of incubation (Figures 1, 30.61 ± 9.77 versus 97.15 ± 27.80, *p* = 0.003 at 0 h and 8.21 ± 1.92 versus 39.57 ± 11.86, *p* = 0.006 at 40 h) (the unit of bacterial counts was 5 × 10^2^ CFU/cm^2^). Meanwhile, Bio-Kil socks from diabetic patients showed significantly lessened bacterial count at 40 h of incubation (Figures 2 and 3 0.76 ± 0.99 versus 25.05 ± 6.99, *p* = 0.003) and revealed a reducing trend of bacterial count in Bio-Kil socks than control socks (4.98 ± 1.89 versus 72.75 ± 37.26, *p* = 0.068). Similarly, Bio-Kil socks from healthy individuals reflected a significantly smaller mean bacterial count than control socks (Figures 3, 56.24 ± 18.45 versus 121.56 ± 41.42, *p* = 0.016), and a declining trend of mean bacterial count was discovered comparing with control socks at 40 h of incubation (12.67 ± 3.56 versus 54.09 ± 22.56, *p* = 0.060).

### 3.3. Safety and Side Effects

There were no reports of contact dermatitis or other adverse effects during the period of research. Furthermore, none of the participants who wore Bio-Kil socks complained with any discomfort.

## 4. Discussion

Diabetic ulcer is a severe complication of diabetes and has always been a problem in real-world practice. The prevention of diabetic foot complications requires a great deal of effort [9]. Numerous studies focused on the improvement of footwear for the prevention of diabetic foot. A variety of socks was designed to significantly reduce peak plantar pressure [10–12]. In some RCTs, the therapeutic footwear could lessen the rate of recurrent ulcer over a one-year period of follow-up [13]. Nevertheless, the possibility of having a footwear with antibacterial activity had not yet been developed for patients with diabetes on the purpose of prevention of diabetic foot.

The Bio-Kil nanotechnology was a promising bactericidal technology that could be applied to any textiles for environment with hygienic needs. The Bio-Kil nanotechnology has been successfully induced to ICU environment or clothes for ICU nurses, and its bactericidal efficacy has been proved after certain investigation [14]. However, this nano-based technology had never been used in footwear for patients with diabetes until our research. In addition, the antibacterial spectrum of Bio-Kil-treated textiles has not yet been studied.

The part of experiments with bacterial strains was designed on the base of clinical practice. In diabetic foot with clinically infected wounds, a tissue specimen for culture was routinely obtained. Superficial and limited foot ulcer infections are usually caused by aerobic Gram-positive cocci, especially *S. aureus*. Chronic and severe foot ulcer infections are often polymicrobially caused, with aerobic Gram-negative rods and anaerobes accompanying the Gram-positive cocci [15–18]. Therefore, we chose *S. aureus* and *E. coli* to be representative of Gram-positive and Gram-negative bacteria, respectively.

Since it was the first time that socks were innovatively treated with Bio-Kil nanotechnology, the bactericide activity was required to be evaluated. In the experiments with bacterial strains, we compared the bacterial counts in socks treated with Bio-Kil and standard shop-bought socks as a true control condition. Bacterial counts diminished in Bio-Kil-treated socks comparing with control socks in all *S. aureus* strains at all incubating durations. In *E. coli* strains, bacterial counts declined in socks treated with Bio-Kil than control socks in five out of six pairs of samples inoculated with ESBL-negative *E. coli* strains, and two out of six pairs of ESBL-positive *E. coli* strain inoculating samples showed lower bacterial counts in Bio-Kil-treated socks than control socks. These results confirmed that Bio-Kil treatment in textiles could result in a self-disinfecting surface with antimicrobial activity, but the activity was stronger for Gram-positive bacterial strains compared with Gram-negative bacterial strains, which indicated that the antibacterial function was not broad-spectrum.

The reason of the phenomenon that Bio-Kil-treated textiles had stronger bactericidal efficacy in Gram-positive bacteria might be associated with the difference of cell wall structure for Gram-positive and Gram-negative bacteria. Gram-positive bacteria has a single-lipid membrane surrounded by a thick layer of cell wall (30–100 nm) composed of peptidoglycan, while the cell wall of Gram-negative bacteria consists of a thin layer of peptidoglycan (2–10 nm) and an outer membrane which contains lipid bilayers and lipopolysaccharides on its outer surface [19]. Since Bio-Kil molecules implement its bactericidal function through damaging the membrane structure of microorganisms with its electrical charge, it could be postulated that the out membrane structure of Gram-negative bacteria might produce certain impairment in the bacterial killing activity of Bio-Kil nanotechnology. Further study that concentrated on the bactericidal mechanism of Bio-Kil molecules could confirm this assumption.

In the part of experiments that volunteers are involved, thirty-one patients with type 2 diabetes and thirty-one healthy participants were enrolled. The study was designed in a single blind, control manner using custom-made control socks in a different color and the same textile with Bio-Kil socks but without treatment of Bio-Kil. Our results demonstrated a significant reduction in bacterial counts in Bio-Kil-treated socks comparing with control socks in the statistical analysis of all participants. The tendency of bacterial count decline was also observed in both diabetes group and healthy participant group, which illustrated that the bacterial killing activity was efficient for both diabetic patients and healthy individuals and indicated that Bio-Kil socks may play a part in prevention and multifaceted treatment of diabetic foot. The footwear showed its feasibility in the prevention of diabetic foot along with other kinds of treatment.

Still, our study has limitations. A main limitation is the lack of assessment of the effect of Bio-Kil on other diabetic foot-associated pathogens, including anaerobes and fungi (e.g., *Candida albicans*) [20, 21]. Furthermore, the sample size was relatively low. Theoretically speaking, an RCT will provide a strong evidence for the bacterial killing efficacy of the sock. However, in terms of availability, the socks used as part of a comprehensive prevention package would be more feasible, which could be further studied. Further work is also required to validate that Bio-Kil-treated textiles has stronger bactericidal efficacy in Gram-positive bacteria due to the different cell wall structure of Gram-positive and Gram-negative bacteria.

In conclusion, foot socks using Bio-Kil bactericidal nanotechnology efficiently reduce bacterial counts in both diabetic patients and healthy individuals and might exert stronger efficacy in Gram-positive bacteria.

## Figures and Tables

**Table 1:** Bacterial counts in Bio-Kil socks and control socks in bacterial strain experiments.

Bacterial counts (5 × 10^5^ CFU/cm^2^)	Bio-Kil socks (0 h)	Control socks (0 h)	Bio-Kil socks (8 h)	Control socks (8 h)	Bio-Kil socks (48 h)	Control socks (48 h)
15B253 (ESBL− *E. coli*)	25^∗^	26	45^∗^	221	22^∗^	60
ATCC25922 (ESBL− *E. coli*)	8^∗^	26	18^∗^	26	2.3	1.1
15B254 (ESBLs+ *E. coli*)	33	13	58	47	13	8.3
ATCC35218 (ESBLs− *E. coli*)	35^∗^	44	44	30	0.06^∗^	14
15B190 (MSSA)	9^∗^	28	2.6^∗^	140	2^∗^	57
ATCC29213 (MSSA)	36^∗^	41	0.79^∗^	14	0.23^∗^	28.4
15B183 (MRSA)	14^∗^	28	4.4^∗^	142	0.4^∗^	4.9
ATCC43300 (MRSA)	59^∗^	68	4.8^∗^	132	0.3^∗^	34

^∗^Bacterial counts were reduced in Bio-Kil socks compared to control socks.

**Table 2:** Baseline data of diabetic patients and healthy individuals.

	Diabetes group (*n* = 31)	Healthy individual group (*n* = 31)
Sex category (male/female)	11/20	14/17
Age (y)	56.6 ± 13.1	28.0 ± 4.5
History of diabetes (y)	9.5 ± 6.7	None
History of diabetic peripheral neuropathy (*n*/%)	13 (41.9%)	None
History of peripheral artery disease (*n*/%)	4 (12.9%)	None

## Data Availability

Data of the manuscript is repeatable and is available after the permission of the corresponding author.
